# How Useful Are Existing Protocols in the Quick Assessment of the Welfare of Semi-Feral Horses? Pilot Study on Konik Polski Horses Living in the Forest Sanctuary

**DOI:** 10.3390/ani14010008

**Published:** 2023-12-19

**Authors:** Aleksandra Górecka-Bruzda, Marta Siemieniuch, Léa Lansade, Christina R. Stanley

**Affiliations:** 1 Department of Animal Behaviour and Welfare, Institute of Genetics and Animal Biotechnology, Polish Academy of Sciences, 05-552 Magdalenka, Poland; 2 The Research Station of the Institute of Animal Reproduction and Food Research, Polish Academy of Sciences, Popielno, 12-222 Ruciane-Nida, Poland; m.siemieniuch@pan.olsztyn.pl; 3 Unité Physiologie de la Reproduction et des Comportements, Institut National de Recherche pour l’Agriculture, l’Alimentation et l’Environnement, Centre National de la Recherche Scientifique, Institut Français du Cheval et d’Equitation, F-37380 Nouzilly, France; lea.lansade@inrae.fr; 4 Animal Behaviour & Welfare Research Group, Department of Biological Sciences, University of Chester, Chester CH1 4B, UK; christina.stanley@chester.ac.uk

**Keywords:** welfare protocols, semi-feral horses, Konik polski horses

## Abstract

**Simple Summary:**

The welfare of horses has been studied mostly for domestic horses used for human leisure, sport, or work. The welfare of free-living horses has only rarely been addressed. The aim of the study was to check the feasibility and usefulness of two welfare protocols developed for free-living horses: a prototype of a welfare assessment template (WAT) for Carneddau semi-feral ponies and the IFCE/INRAE Horse Welfare Protocol for welfare inspection. They were used in July/August 2022 and April 2023 to evaluate welfare of a pilot population of nineteen free-living Konik polski horses. The indicators of both protocols were scored high or satisfactory. Only the body condition was lower in early spring than in summer. Our study confirmed the feasibility of most of the WAT and IFCE/INRAE welfare indicators in semi-feral horses. Some adaptations are needed for in-field application. Merging both protocols, validation and considering positive welfare indicators would increase the applied potential for welfare assessment in semi-feral horses.

**Abstract:**

Scientifically validated and standardised methods for the evaluation of the welfare of free-living horses are urgently needed by both the owners and managers of these populations and those responsible for implementing national welfare legislation. The aim of the study was to test the feasibility and usefulness of two welfare protocols that could be applied to semi-feral populations: a prototype of welfare assessment template (WAT) for Carneddau semi-feral ponies and the IFCE/INRAE Horse Welfare Protocol. Additionally, the body condition scale designed by Henneke (BCS-H) was employed. The study took place in July/August 2022 and April 2023 to evaluate the welfare of a pilot population of nineteen semi-feral Konik polski horses. The horses scored high or satisfactory under indicators across both protocols; only body condition scores were significantly lower in early spring (BCS-WAT: 1.11 ± 0.57; BCS-H: 3.84 ± 1.17) than in the summer (BCS-WAT: 1.58 ± 0.61; BCS-H: 5.63 ± 1.01). Our study confirmed the feasibility of utilising most of the WAT and IFCE/INRAE welfare indicators in semi-feral horses. Some adaptations, such as considering validation of scales, positive welfare indicators and animals’ free-choice of conditions, have been suggested for future in-field application.

## 1. Introduction

An awareness of the importance of living and working standards for domestic animals has contributed to increased public and institutional interest in the welfare of horses. However, since the public approach to this issue may range from excessively compassionate to complete objectification of animals [1], scientifically validated and standardised methods for the evaluation of horse welfare are a key requirement for objective welfare standards to be upheld.

Many welfare assessment protocols for horses are currently available. These include the AWIN Welfare Assessment Protocol [2]; Welfare Monitoring System, Assessment Protocol for horses, Wageningen UR Livestock Research (2011) [3], EARS—Equid Assessment, Research and Scoping [4], SEBWAT—Standardised Equine-Based Welfare Assessment Tool [5], WAG—Welfare Aggregation and Guidance Tool [6], HWAP—Horse welfare assessment protocol [7] and the IFCE/INRAE Horse Welfare Protocol [8]. These have been developed specifically for breeding, sport, leisure or working horses. Two of them, the AWIN Welfare Assessment Protocol and the IFCE/INRAE Horse Welfare Protocol, were mostly based on scientifically validated indicators and made publicly available to be used in practice via free smartphone applications. Most of the welfare assessment protocols were designed and tested on horses that are routinely handled and whose environment is heavily controlled. However, according to our knowledge, only two welfare assessment protocols have been designed specifically for feral (wild) or semi-feral (free-living but owned) horses: the Ten-Stage Protocol [9,10] and a welfare assessment template that was developed using a population of semi-feral Carneddau ponies [11].

Despite their relatively natural lifestyle, the welfare of free-living horses can be significantly impacted by both the environment and by human impacts. Feral and semi-feral horses live year-round in a relatively natural environment and occur worldwide [12]. Observations of feral horses living in naturalistic conditions provide knowledge about all aspects of the behaviour of the species [12], which is often used as a welfare reference for horses living in human-controlled conditions. However, it has been shown that free-living horses can also suffer from decreased welfare [13,14]; here we define animal welfare according to [15]: ‘welfare corresponds to the absence of negative subjective emotional states, usually called “suffering” and probably with the presence of positive subjective emotional states, usually called “pleasure”’. Poor welfare could occur in free-living horses due to their inability to meet their core nutritional requirements, for example, when horses suffer a shortage of forage due to the season (in winter or due to drought, [14] or due to overgrazing [16]. At the opposite end of the spectrum, obesity can be a frequent problem in native horse breeds in the high grazing season; this could be another welfare issue in free-living horses [17,18]. Water availability can also be an issue. Feral horses usually drink from natural waterholes such as streams, rivers, lakes, and ponds but also from man-made watering installations such as reservoirs and canals. Drought or the scarcity of water can make them travel to waterholes up to 70 km away to satiate their thirst [19]. The quality of water can sometimes be poor as horses trample the borders of natural waterholes making them swampy and dirty. Feral and semi-feral populations living in rocky or swamp environments are also at risk of injuries or fatal incidents due to rough terrain [13,14]. They can also sustain injuries as a consequence of fights between stallions and stallion harassment [20], infected insect bites [21], high levels of parasitism [22,23,24] or predator attacks [25,26], and hoof and leg deformations causing lameness [17,18]. Human impacts can also affect the welfare of free-living populations, for example via collisions with cars [27].

The Ten-Stage Protocol [9,10] and the welfare assessment template for Carneddau semi-feral ponies [11] addressed the issue of welfare in free-living horses. The Ten-Stage Protocol [9,10] was developed on the basis of the “Five Domains” model: nutrition, physical environment, health, behavioural interactions and resulting mental state. The ten-stage protocol involves, in addition to direct observation, data from camera traps [10]. This kind of assessment needs investment in electronic devices and is time-consuming in terms of data gathering and analysis. In cases when horses have very similar coats without markings, they cannot be identified with certainty from photos or videos. While the Ten-Stage Protocol [9,10] based on the five domains of welfare was developed for wild, feral horses that are difficult to observe from a close distance [14], semi-feral (owned) horses are much easier to approach and observe directly. In Europe, free-living horses are usually owned (semi-feral) and maintained in common lands or sanctuaries, e.g., [25,28]. They are kept in the semi-feral state either as a legacy of longstanding agricultural tradition and local breed protection [25,28] or because they were introduced to naturalistic environments as a result of projects for biodiversity maintenance [29,30] or rewilding [16,30]. According to EU Law [31] all horses must be monitored by their owners, so they are habituated to visitors. Severe injuries or other incidents in semi-feral horses may need veterinary or management intervention. In such cases, a systematic welfare assessment may prevent fatal incidents or alarm the owners or local responsible authorities at an earlier point. 

Konik polski horses are a native Polish breed, which was created with the intent of reintroduction to a primaeval forest environment, meaning they must have the ability to cope with difficult conditions [13]. Although present Koniks, like many pony-type breeds, are also maintained in traditional stables and used for leisure riding, still they are bred mostly with the aim of retaining the ability to survive in harsh environments. Since they successfully fulfil the criteria of robustness and good health in various environmental conditions, they are used in many reserves in Europe for the maintenance of biodiversity and landscapes [13].

Like other equines in Europe, Koniks living in reserves have to be regularly monitored. Although they are inspected by the breeding manager on a daily basis, we were interested in testing a rapid and repeatable method of welfare assessment that any trained observer could use for the periodic inspection of free-living Konik horses living in Polish reserves. As the WAT prototype template for Carneddau semi-feral ponies [11] is based on direct observations of animals and since Koniks roam over a limited area, are habituated to humans, and can be directly observed, we chose to test the Carneddau welfare assessment template (WAT). This protocol was developed for the assessment of the welfare of free-ranging ponies in the Carneddau mountains, United Kingdom, to address concerns raised by the public regarding a lack of supplementary feeding and shelter for horses [11]. The indicators included in this protocol are repeatable and were found to have good levels of inter-assessor reliability [11].

As Koniks are maintained in outdoor conditions, we decided also to use the validated protocol developed for domestic horses living inside or outside stables: the IFCE/INRAE Protocol Cheval Bien-Etre [8]. The core of this protocol is the AWIN horse protocol which consists of reliable and validated welfare indicators [2]. It was used in a recent study [32] to assess the welfare of horses in an outdoor group housing system, the “parcours”. Importantly, the IFCE/INRAE protocol involved the evaluation of at least three indicators of negative emotions, likely to indicate reduced well-being: stereotypies, signs of indifference towards the environment (including a “withdrawn” posture, or signs of apathy) and aggressiveness towards humans. As defined by [33,34], the “withdrawn” posture was characterised by the horse displaying an unusual posture, standing with eyes wide open, a stretched neck (open jaw-neck angle), with the neck held at a similar height to the back, an unusual gaze, head and ears fixed, ears focused mostly backwards, and showing indifference towards environmental stimuli (visual, tactile and auditory). The ears being directed backwards whilst the horse was eating was also assumed to indicate discomfort or poor welfare. The evaluation of the reliability of these indicators, included in the IFCE/INRAE protocol, was based on studies by Fureix et al. 2014 [33] and Lesimple et al. 2016 [34]. 

The IFCE/INRAE Protocol [8] included several modifications to the AWIN protocol: there is a greater choice of levels for each criterion, more behavioural measures are included (e.g., recording the presence of the “withdrawn” posture), and some parts of the test were adapted to be used outdoors (e.g., human avoidance test). The interpretation of the data were not based on a reference dataset but on recommendations from the scientific literature. Although this protocol was designed to evaluate horse welfare in human-controlled conditions (stables and pastures), the recent use of AWIN protocol, constituting the core of the IFCE/INRAE Protocol, in the “parcours” study proved it may be applied for horses in extensive conditions after some modifications; we, therefore, chose to test its usefulness for welfare monitoring in semi-feral horses in parallel with WAT [11]. 

The aim of this study was to test in the field the feasibility and usefulness of these two protocols for the welfare evaluation of semi-feral Konik polski horses but also in terms of their potential applications to other semi-feral horse populations. We define feasibility as “the capability of being done or carried out” and usefulness as “the quality of having utility and especially practical worth or applicability” (https://www.merriam-webster.com/dictionary, accessed on 18 December 2023). We were also interested in whether these protocols are able to capture the changes in welfare that can occur across seasons in East-Central Europe climatic conditions, so the assessment was carried out in both summer (July/August) and in spring (April). The outcome would be an easy-to-use and feasible tool that could be utilised as a welfare assessment tool by owners, inspectors or veterinarians visiting semi-feral horse populations.

## 2. Materials and Methods

### 2.1. Animals

In July/August 2022 and April 2023, a population of nineteen semi-feral Konik polski horses was sampled. The horses were 9.64 ± 6.98 years old (median 9, interquartile range 4–14, range 1–28). The horses live in a 1600 ha forest sanctuary located in the region of Mazurian Lakes, Popielno peninsula, Poland (53.754556, 21.628361). The habitat consists of deciduous mixed forests and coniferous forests (about 95% of the area), and open grasslands (5% of the area). The horses were free to choose their resting and feeding locations within the sanctuary, and water was available in nearby lakes, canals, and ponds. During winter, when the snow cover stayed for more than one week, the horses were provided with hay. The detailed practices of management of animals in the Popielno sanctuary were described in [13]. 

The horses live in social groups (bands) typical of free-living horses (described in detail in [13]). In 2022, one band consisting of a stallion and six mares (a harem), one harem consisting of a dominant stallion, eight mares and a two-year-old subordinate stallion and one bachelor band consisting of a three-year-old stallion and a one-year-old colt were selected to be sampled in this study. Between 2022 and 2023, the groups split into three harems when a four-year-old bachelor stallion took over three mares from existing harems. The two-year-old bachelor then became a solitary male in the spring of 2023. 

### 2.2. Methods

#### 2.2.1. Preliminary Adaptation of Protocols

To achieve the aim of our study, to evaluate the usefulness of these protocols for inspectors and owners, we first analysed our chosen protocols in terms of feasibility as suggested by the Ten-stage Protocol for assessing welfare in free-roaming horses [9]. Knowing the conditions of Koniks living in the sanctuary, the particular regulations concerning them (preferably that the horses should not be touched), and having in mind the possible short time for welfare assessment in practice, we scrutinised both protocols to keep only the indicators and methods we could use in the field, similar to the “parcours” use of the AWIN protocol [32]. Due to the requirements to maintain the Koniks to be as “wild” as possible, we did not touch the horses, except for marking some horses with a cattle pencil marker (since it was otherwise difficult to record which horses had so far been subject to the welfare assessment). We also did not test the horses with the “human approach test” in either protocol; instead, we used the “human avoidance distance”, i.e., the response of the horse to the presence of the observer or camera operator. The Henneke et al. [35] scale (BCS-H), as modified in the WAT feeding/nutrition scale to only use visual assessment (BCS-WAT) was also applied. For WAT, we also separated the assessment of “resting comfort and high human presence” into “resting comfort” and “high human presence” as we decided that the latter one is already included in the rating of “human disturbance” in the original protocol. We also decided not to use the horse grimace scale (HGS, AWIN [2]); since we had no training in this method, and we were not confident that the potential final users would be able to evaluate it reliably.

The IFCE/INRAE Protocol Cheval Bien-Etre [8] could not be used as a complete tool. Therefore, similarly to the approach taken by Dai et al. [32], we adapted the protocol for our purposes. We omitted the assessment of body condition, as this required physical handling of the horse. Further, the measures related to stabling and riding such as faecal consistency, mouth injuries, the box’s dimension, the quantity of forage per day, and the frequency of turnouts were not used for the welfare assessment of our free-living horses. We also merged “vaginal or penial discharges and prolapsus” with “other discharges” to include anal discharges/diarrhoea.

As directed by the IFCE/INRAE protocol [8], every horse was observed for three seconds at thirty-second intervals for a five-minute observation period, leading to ten sampling points. The occurrence of any stereotypical behaviour, “withdrawn” posture or aggression towards humans during these sampling points was recorded. If any stereotypical behaviour or aggression towards humans was observed, or five successive “withdrawn” postures, welfare was classified as unsatisfactory [8]. To record ear position, the same sampling regime was used, but this had to be during five minutes of the horse being engaged in foraging behaviour; ear position was observed for three seconds at thirty-second intervals for this five-minute observation period. Three potential ear positions could be recorded: both ears directed forward, both ears directed backwards or one or both ears in an intermediate position. If more than two successive sampling points included backward ear positions, welfare is considered unsatisfactory [8]. Other IFCE/INRAE [8] indicators were used as defined in Table 1.

For the purposes of the present study, most IFCE/INRAE indicators were rated as “1” to represent “satisfactory” welfare or “0” to represent “unsatisfactory” welfare, with some indicators including an intermediate stage of “0.5” denoting “intermediate” welfare. We used the original scores from the WAT assessment for its indicators; these vary between 0 and 3, as justified by Harley et al. [11]. WAT indicators with option “0” were deemed a more severe level of welfare compromise. The indicators retained in the final assessment from both protocols, and explanations for the scores assigned to these, are presented in Table 1.

#### 2.2.2. Observations

On the observation days, the horses were located by visiting the areas where they are regularly sighted and with the help of the person responsible for monitoring the horses’ health on a regular basis (the breeding manager, MS). The identification of individual horses was confirmed by the breeder. The horses were observed during two discrete data collection periods so that seasonal variation in BCS could be assessed. In summer 2022, the 19 horses were individually observed across four days (27 July 2022, 30 July 2022, 1 August 2022 and 2 August 2022) since the bands could not all be located on the same day. The mean temperature, cloudiness, and wind speed were 21.3 ± 3.1 °C, 53.1 ± 4.74% and 13.7 ± 5.21 km/h respectively. In 2023, all bands could be located, evaluated and assessed on the same day (18 April 2022). On this day, the temperature, cloudiness and wind speed were 15 °C, 50% and 12 km/h, respectively.

**Figure 1:**
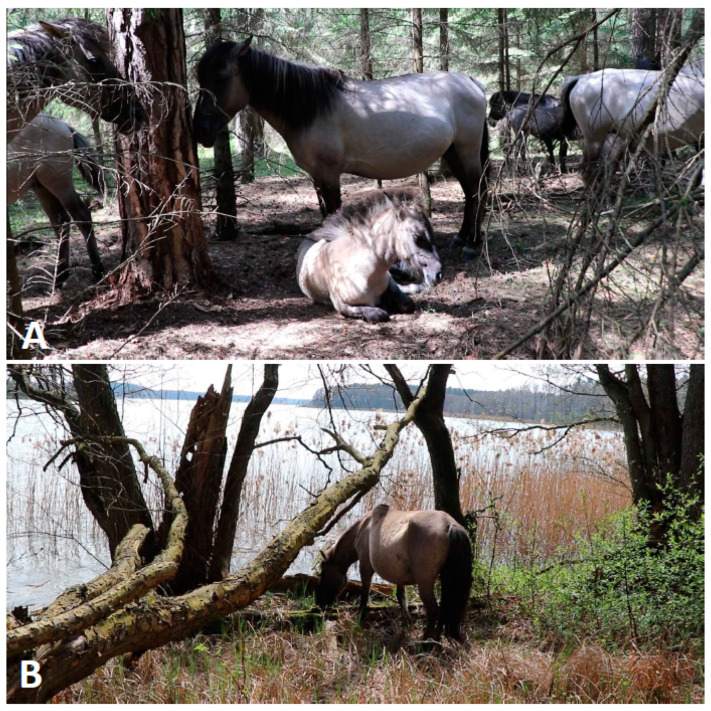
Two examples of resting locations that made implementation of some welfare indicators challenging. (**A**) Close grouping within densely forested areas sometimes made observations of individuals from all angles impossible. (**B**) At other times the horse’s choice of location meant that the observer was not able to carry out observations from directly in front of the horse. (Photos by Michał Bruzda).

Two people were involved in the gathering of the behavioural data, the camera operator (MB) and the observer (AGB), a researcher experienced in welfare assessment. The horses were observed from a distance that we determined did not affect their behaviour (not closer than three meters, unless the horse voluntarily approached the humans). If the horse approached the humans, it was marked on the neck with a cattle marker to differentiate successfully observed animals. For archiving and identification purposes, the focal horse was video-recorded for five minutes by the camera operator, first in lateral view from one side, mainly the one illuminated by the sun, and then by zooming in on the eyes, nose, and hooves. At the same time, the observer assessed all welfare indicators by observing the horse from all sides, where possible. Occasionally, the horses grouped so tightly in densely forested areas that they could not be safely approached and seen from all sides (Figure 1A). Also, at times, hooves could be hidden in the grass and could not be scored, thus in 2022 only 17 and in 2023 only 18 of 19 horses had their hooves evaluated. When the horses started to move, they were followed until the focal animal stopped or started to graze to complete the five minutes of welfare assessment, except for cases where they chose a way that could not be crossed by humans, meaning they were observed on another day. The environment was consistent for all horses, so these indicators were evaluated at the beginning of the observation of a group of horses. During the five-minute behavioural observations, the researcher checked for injuries or insect bites. At the end of the observation period, these were recorded together with the condition of the coat, mane and tail, hooves (if visible), and body condition was also scored. Since most horses were in bands of four to nine individuals, social interactions occurring between any group members were recorded opportunistically if these occurred during any focal observation; this allowed a longer period of time to assess whether horses had the opportunity for social interactions.

#### 2.2.3. Statistical Analysis

All analyses were carried out using the SAS 9.4. statistical package (SAS Inst., Inc., Cary, NC, USA). Due to non-parametric distributions of the scores, the effect of season/year (summer 2022 and spring 2023) was tested with a Sign rank test for related measures. Analyses were not performed if there was no variability in the measure. To test individual consistency in body condition in the population across seasons we carried out Spearman’s correlations between the BCS measurements in 2022 and 2023.

## 3. Results

### 3.1. Protocols’ Feasibility

Not all of the selected indicators (Table 1) could be measured for all individuals. For example, the WAT indicator “faecal consistency” could not be assessed for all horses. Defaecation events were sporadic, and with a restricted observation time, only a few defaecations were observed. In addition, not all indicators in the IFCE/INRAE protocol could be used in practice. This protocol directed the observer to record the occurrence of stereotypies, ”withdrawn” postures, and aggression towards humans during three-second sampling points at 30 s intervals over a five-minute period in non-foraging horses. Ear posture was then to be sampled using the same protocol but during five minutes of consistent foraging. In practice, horses regularly changed their behaviour and consistent foraging periods were not always observed. Also, positioning the observer in front of the horse to note ear position was not always possible due to close individual distances between horses, dense forestation, or the spatial environment (Figure 1A,B). We, therefore, discarded the ear position indicator, and observed for the occurrence of stereotypical behaviour, a “withdrawn” posture or aggression towards humans over a five minute period, regardless of the behaviour in which the horse was currently engaged. The remainder of the indicators from the original protocols could be used (Table 2 and Table 3).

### 3.2. The Effect of the Season

**Figure 2:**
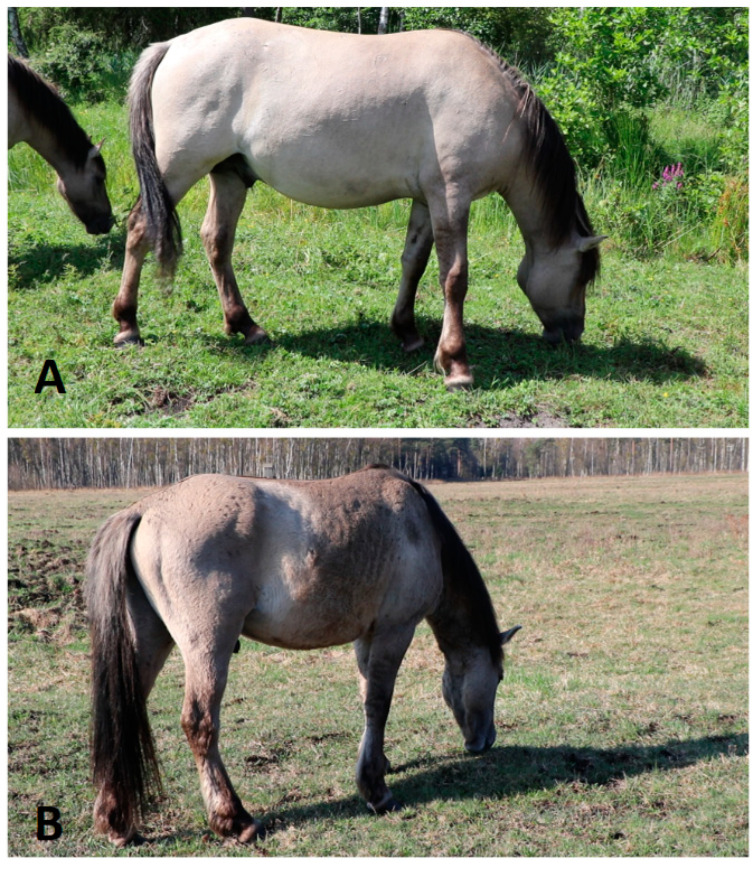
Seasonal changes in body condition in the subordinate stallion (**A**) Summer 2022, (**B**) Spring 2023 (photos by Michał Bruzda).

In general, both protocols confirmed a high level of welfare in the Koniks that were assessed; most of the time, they were assigned the most positive scores for each indicator (Table 2 and Table 3). However, some signs of poorer individual welfare were recorded, as indicated. Season only had an effect on body condition (summer 2022: BCS-H, 5.63 ± 1.01, range 2–6; BCS-WAT, 1.58 ± 0.61, range 0–2; spring 2023: BCS-H, 3.84 ± 1.17, range 2–7; BCS-WAT, 1.11 ± 0.57, range 0–2) when using the scales from [35] and [11] studies (Table 2, Figure 2A,B). The season, in general, had no effect on other indicators within either protocol.

### 3.3. Welfare Assessed with WAT Protocol—Qualitative Analysis

Both the environmental and individual WAT indicators of welfare were scored as satisfactory or received maximal scores, except for BCS for one geriatric mare, in both seasons (Table 2). Water was available from canals and lakes, so the cleanliness was not comparable to spring-fed lake or stream waters. Even if the horses were located at some distance from water sources (maximum 1 km from the lake) they were known to visit it regularly (personal observation, AGB). Human disturbances occurred due to the location of the sanctuary in the frequently visited tourist region of the Mazurian Lakes. The highest intensity of tourism-related activities occurs mainly in July and August. It involves car traffic on the main road, biking on the road, and on a bike trail located in the western part of the reserve; otherwise, there is only sporadic pedestrian activity in the deep forested areas where the Koniks range. In the summer months, there are also boats moored on the border of the reserve. The Koniks were occasionally observed approaching tourists for treats on the road and at the lake border (AGB personal observation). However, this does not occur outside of the tourist season, and even then, the horses spend most of the time in the forest, out of human view and potential disturbances. The road is therefore the only potential man-made hazard, and the horses can easily avoid it.

The horses have a good choice of locations where they can rest and lie down. There were no wet, dirty patches on their coats, indicating they were lying on suitable surfaces. No thermal discomfort was scored by WAT, the horses could always move to shaded places (e.g., Figure 1A). No horse presented lameness, which was evidenced by opportunistic observations of the whole group during video recordings of individual horses. The mane, tail, and skin conditions were adequate for the season, except for geriatric horses, and one horse showing skin irritation due to insect bites in the summer. One clear ocular discharge was observed in spring 2023 for the same mare presenting an insect allergy; otherwise, no other discharges were observed. Across all 38 observations, coughing was only recorded once, in a two-year-old bachelor. Two horses presented small (less than 5 cm), non-bleeding, non-swollen, healing wounds. All horses stayed in their social group (Figure 1A and Figure 2A) except for the previously mentioned young bachelor colt that was found alone in the spring of 2023 when his companion started his harem-leading role. The bachelor was not accepted as a subordinate stallion either by his previous companion or by two other harem stallions. All horses accepted the close presence of humans, presenting no fear or flight response. Some of them voluntarily approached and sniffed the observer and video operator.

### 3.4. Welfare Assessed with IFCE/INRAE Protocol—Qualitative Analysis

Almost all indicators of the IFCE/INRAE protocol were on the “satisfactory” (1) level (Table 3). Detected problems included coughing, coat and skin condition, injuries, ocular discharge, and water cleanliness, as described above for the WAT. These indicators were also covered by the IFCE/INRAE protocol, evidencing compatibility with the WAT descriptors. Otherwise, stereotypies, aggression towards humans, human avoidance, thermal stress, swollen joints, other discharges, and lameness were not observed. Moreover, as the hooves could be seen in almost all horses, this indicator was retained in the final version of the protocol. One case of aggression towards a human was observed, but not recorded (due to this being initiated by a non-focal individual), when the camera operator following the focal mare during recording was lightly kicked by another one. Observed social interactions within bands involved affiliative and mild agonistic behaviours typical in horse bands. However, the solitary bachelor colt, which tried to maintain contact with other horses by approaching other bands, was mostly chased away by harem stallions. 

### 3.5. Body Condition Scores in Both Seasons

The horses showed consistency in relative body condition across the seasons as shown by the positive correlation between their scores in summer 2022 and spring 2023 for BCS-WAT (r_s_ = 0.58, *p* = 0.01). BCS-H was not significantly correlated across seasons (r_s_ = 0.18, *p* = 0.46). 

## 4. Discussion

### 4.1. Usefulness and Feasibility of Protocols Tested 

As it was originally developed for free-living horses, the Harley et al. [11] WAT proved very useful for the assessment of welfare in our sample. It considered environmental aspects that were not specified in other protocols, such as potential human-made hazards. The WAT was originally developed to observe the horses without any time constraints but was useful and feasible in our field conditions, even in the 5 min session that inspectors are frequently restricted to. Since semi-feral horses are usually found in groups, in practice the researchers observed social behaviour opportunistically for a much longer period, since other group members’ interactions could be recorded at the same time as focusing on each individual for five minutes.

Using the IFCE/INRAE protocol, developed for stabled and pastured horses that are used mainly for riding, most indicators could be measured; however, some were less useful and feasible in naturalistic conditions. Specifically, the recommendation in the IFCE/INRAE protocol to observe the horses twice (when feeding and not feeding) would extend the observation time; this would be impractical for rapid welfare assessment by welfare or veterinary inspectors. However, a 5 min observation per horse appeared to be a sufficient length of time to record the minimum information required regarding the physical and mental state of the horse for a basic measure of its welfare to be determined. In addition, the IFCE/INRAE protocol includes an intermediate level for some items, in contrast to the original AWIN protocol. It seems beneficial to incorporate this intermediate level into the future protocols for a broader range of criteria to enhance flexibility by scoring intermediate states. If this were to happen, the validity of the scale used for these indicators would need to be assessed. 

Since both protocols contained several identical indicators (WAT: “Water availability”, “Resting comfort”, “Thermal comfort”, “Mobility”, “Skin/mane/tail and coat condition”, “Ocular/nasal discharges”, “Coughing”, “Wounds and swellings” and IFCE/INRAE: “Water—availability”, “Resting comfort”, “Thermal stress”, “Lameness”, “Coat condition”, “Ocular/nasal discharges”, “Coughing”, “Injuries”, “Joint swollen”), we suggest these protocols can easily be combined to give a clearer assessment of welfare in semi-feral horses. The following indicators: “Mobility/Lameness”, “Skin/mane/tail and coat condition”, “Ocular/nasal discharges”, “Coughing”, “Wounds and swellings” “Thermal stress”, “Coat condition”, “Ocular/nasal discharges”, “Coughing”, “Injuries”, “Joint swollen” were easy to assess using both protocols. Nonetheless, the assessment of some indicators posed more difficulties. Below, we discuss potential issues with the definitions and during the assessment of welfare with the indicators used in the studied pilot population.

### 4.2. Welfare Indicators: The Definitions and Potential Issues with the Application

We found that the definitions of some indicators were not easy to understand and implement in the field, or required different interpretation according to the context, for example, how to assess “water availability”. We observed horses in the forest where there was no waterhole, but there was one a few hundred metres away; we only realised later that this further waterhole should be the one assessed. Also, in another location, “water cleanliness” was better in a lake 1 km from the location of the band, where the horses could easily go, but instead, they drank from the canal situated in the grazing area, meaning this was scored low but obviously preferred. In addition, since the WAT was developed for horses moving freely in naturalistic yet also human-impacted areas, man-made hazards such as hazardous fencing materials, telephone wires, roads, or mountain bikers were included as potentially negatively impacting the welfare of semi-feral horses. However, since horses are domesticated animals, in many cases, they are able to habituate to or avoid such hazards. It could therefore be argued that despite the presence of some of these potential hazards, welfare is not compromised given the horses’ ability to avoid these in their daily life. Perhaps this indicator could be adapted to include whether this choice to avoid the hazard is there; this would differentiate between a population that lives alongside a road yet rarely crosses it and one that must cross a busy road on a daily basis to move between watering and grazing areas.

“Mobility” (WAT) and “lameness” (IFCE/INRAE) consider a similar aspect of welfare, but the WAT protocol proposed an intermediary score for a lame horse that can follow and stay with the band while the IFCE/INRAE protocol did not accept lameness as an acceptable welfare state under any circumstances. This is understandable since the latter protocol was developed for domestic horses that are under human care. Free-living horses can sometimes wear down their hoof horn resulting in cracks in the hoof wall that can be deep and cause pain and lameness [13,36]. Usually, these cracks heal as the horn grows, and the lameness disappears, meaning lameness can be a temporary impediment as opposed to indicating a severe injury. Nevertheless, any degree of lameness can impact the horse’s potential flight speed which, given the recent return of wolves to European forests and nearby grazing areas for horses [25,26], could put lame horses at much higher risk of predator attacks. We therefore suggest that the presence of lameness could be a factor that is weighted according to the risk of predation in a population. 

The “skin/coat/mane and tail” indicator can be a useful welfare indicator in some seasons, but in others this indicator might be more difficult to reliably assess. While summer coats allow easy inspection of injuries, parasites and body condition, the winter coat, especially in primitive breeds of horses living in colder regions [37,38], could effectively mask small wounds or poor body condition. It is therefore important that welfare is assessed across different seasons, and that welfare assessments carried out in winter months are treated with appropriate caution.

The IFCE/INRAE protocol’s indicator “possibility for social interactions” quantifies an important aspect of welfare, yet this could need more nuanced interpretation in some instances, such as for one of the colts included in this study. Horses are highly social animals; they have a strong urge to remain near conspecifics even if they are subordinate and are the receivers of agonistic behaviour from other horses, e.g., [39]. In the current study, one colt was able to bond with another solitary stallion in one season, but in the next season, his companion had left to form his own harem, leaving him alone. At this point, the colt began to voluntarily approach humans; this type of behaviour has previously been reported for individually kept foals [40] and could be viewed as abnormal. In most free-roaming populations, there would be the option for dispersed colts such as this to join a bachelor band; however, there were no other solitary stallions in this population at the time of this study. This colt, therefore, did not, in this second season of data collection, have an opportunity for positive social interactions; he attempted to do so, but was constantly driven away. There could be two courses of action in this case. The colt could be removed from the population due to a lack of positive social interactions indicating potentially compromised welfare; however, it would be highly likely that this colt would then be housed without the opportunity for social interactions, as is routine practice in the management of domestic stallions [41]. A better action might therefore be to leave the colt in this situation with the expectation that his solitude was temporary and, at some point, he would form a harem. This is an example where human decisions regarding the optimal welfare for horses may change according to individual circumstances or challenges. 

Positive indicators of welfare should be a useful addition to protocols. Like most existing welfare protocols for horses, the WAT and IFCE/INRAE protocols mostly focus on negative aspects of horses’ physical and mental conditions. As proposed by Harvey et al. [14], the positive mental states related to the satisfaction of physiological and social needs should be included in future protocols, according to Duncan’s [15] definition of welfare. Although we observed the horses as satiated, relaxed, socially satisfied, and able to choose preferred sites for resting, foraging and drinking, this would allow a more in-depth assessment of welfare [14].

### 4.3. The Welfare of Koniks as Assessed by Protocols

**Figure 3:**
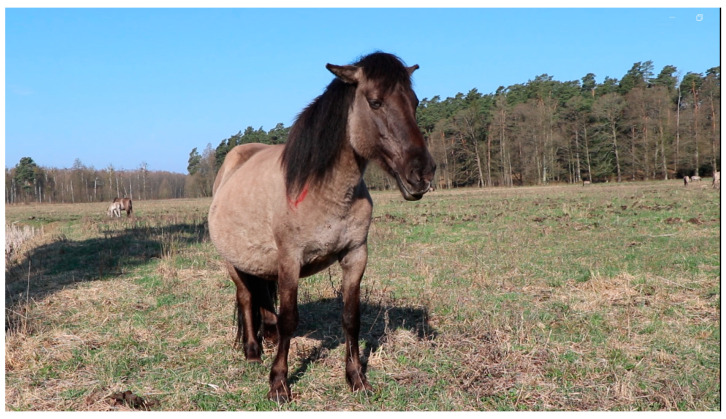
This shows the same band as the photo in Figure 1A; this photo was taken in spring 2023 when a much higher inter-individual distance was apparent. The red mark on the horse’s neck was drawn by the observer using a cattle marker to indicate that this individual had been assessed. This is carried out as it can be difficult to distinguish between individual horses due to a lack of naturally occurring markings. (Photo by Michał Bruzda).

In general, the welfare assessment of the pilot population of Konik polski horses according to both protocols indicated a high level of welfare across most indicators. Our study confirmed earlier observations that the stereotypies frequently observed in stabled horses are not performed by horses living in natural conditions [42], although some abnormal begging behaviour was observed. No ”withdrawn” posture, as described by Fureix et al. [33], was presented by the horses; however, they were sometimes observed to maintain a high individual distance and seemed to be indifferent to environmental stimuli. When resting or pasturing, the horses could group either tightly (Figure 1A) or more sparsely (Figure 3), which was likely to be related to insect harassment on warm days as compared to colder springtime weather [21]. As free-living horses are better able to choose their resting place and inter-individual distance than are domestic horses, due to generally having a much larger area in which to roam, this shows again that horses may choose to separate from and reunite with conspecifics on a fission-fusion basis, perhaps due to environmental fluctuations [43].

The BCS-H and BCS-WAT body condition scales were both able to capture a seasonal effect on body condition. In terms of decreasing welfare for fatter horses, the BCS-WAT may be not as effective in recording fine-scale changes in weight, so the use of the two in tandem gives more information regarding the individual’s true welfare status, where this is feasible (although the original BCS-H requires physical palpation, which is usually not possible for free-living horses). As the BCS-WAT was positively correlated within individuals across seasons, individual horses may have consistent tendencies for better or worse condition regardless of the season [44]. 

The observed decrease in BCS was likely to be related to the change in nutritional content of the horses’ diet due to the lower quality of the forage in winter. Seasonal changes in body condition are observed annually in Konik horses [13]. Despite non-lactating mares occasionally becoming obese in the summer season, only two cases of laminitis have been observed in this population in the last 30 years [13]. Since our study mostly included lactating mares, they were generally only in moderate condition, even in summer.

The environmental conditions of the reserve and the control of the stocking rate by removing almost all foals each year prevent the degradation of habitat and have ensured adequate nutrition for more than 10 generations of Koniks. The reserve also provides unlimited access to water via natural waterholes which is fundamental for health and welfare [45]. Deep canals and swamps on lake borders may, however, be hazardous, and they have caused fatalities in the past [13]. Similar to the Carneddeau Mountains, the Mazurian Lakes are a frequently visited region by tourists. As with other similar reserves or when pasturing on common lands, car traffic can be problematic for horses [27]. Human disturbance can be avoided by Koniks, but feeding the horses with inappropriate treats by tourists (officially forbidden) jeopardises the health of Koniks and encourages them to come near to a seasonally busy road. Moreover, demanding treats may make the horses “pushy” or even aggressive towards humans, as observed for one stallion (personal experience, AGB). Since this may be problematic for human safety, it is preferable that in similar reserves with free human admission, horses maintain a greater distance from humans. 

**Figure 4:**
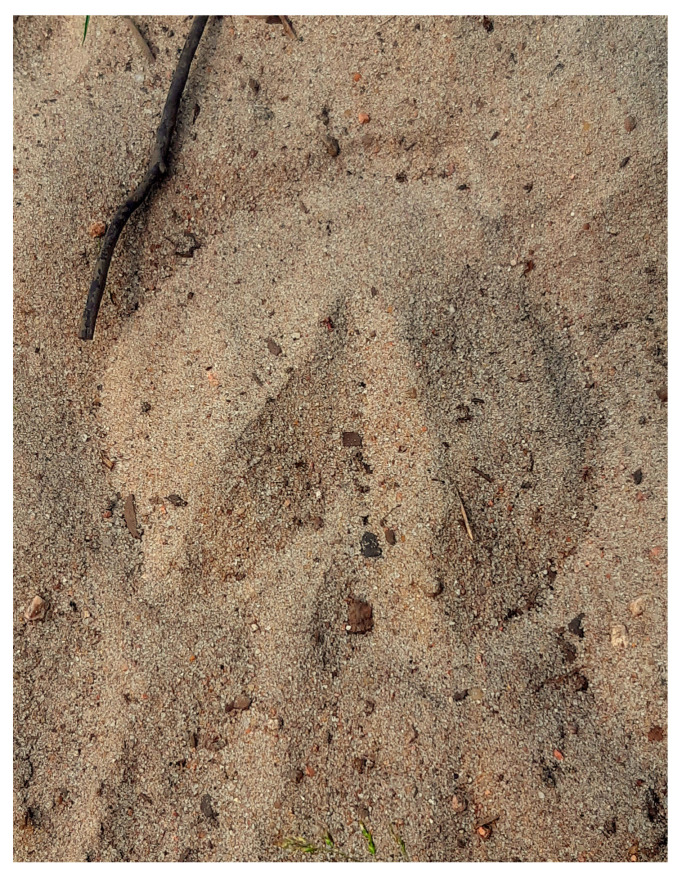
The footprint of the Konik hoof. The Koniks’ hooves are not trimmed and the natural environment in the sanctuary allows for adequate wearing down of the hoof for lameness to be avoided. (Photo by Aleksandra Górecka-Bruzda).

Health problems, such as wounds and discharges, can be observed in free-roaming horses [13,14] but have only rarely been reported in the literature. Studies on hoof health show that feral horses may suffer from laminitis or hoof cracks that provoke lameness or limb deformations [17,18]. Our results show that Koniks sufficiently wear down their hoof during daily locomotion and no lameness was noted (Figure 4), but in other reserves, especially those with a high proportion of wetlands, hoof health may be problematic.

### 4.4. Validity of Welfare Indicators

The WAT indicators have previously been tested in terms of inter-assessor reliability and repeatability over time and were based on validated indicators of welfare [11]. Similarly, the IFCE/INRAE protocol was based on validated indicators from the AWIN protocol [2]. However, a future comprehensive protocol for the reliable evaluation of welfare in semi-feral horses would require validation for all the indicators included, especially where intermediate scores have been added, due to these being applied in a novel context. This study has identified potentially useful indicators and recommended changes where required; the next step would be validation and publication of a ready to use protocol.

## 5. Conclusions

Our study confirmed the feasibility of most of the WAT and IFCE/INRAE welfare indicators in this semi-feral horse population. Most of the indicators included in the final assessment adequately describe the welfare status of the horses. However, some adaptations were needed for in-field application, particularly concerning the definitions of some indicators and their subsequent validation, as well as the observation time (since we assumed that only five minutes per horse could be available for welfare assessments). We suggest that some adaptations, including animals’ ability to select optimal conditions, would be beneficial for the in-field application of updated welfare assessment protocol for semi-feral horses. Also, the assessment of positive aspects of welfare would be beneficial.

To reliably assess and monitor welfare, we recommend long-term monitoring of free-living populations. We would not support making welfare decisions based on individuals’ social situations as assessed during a one-off observation as these often vary across seasons, especially for younger animals dispersing from their natal groups. Since the ongoing experiment in the Popielno reserve aimed to assess the ability of horses to adapt to naturalistic conditions over a long-term period, we have conducted many years of continuous monitoring; we have observed that many health or social issues were resolved over time without intervention. Ten generations of horses have been able to survive and efficiently reproduce in a reserve that does not provide clean spring water or shelters. Although in some cases human intervention is required, this breed is very robust and well adapted to harsh conditions. Nevertheless, regular welfare monitoring is required to ensure an acceptable level of welfare is maintained.

The welfare of the sampled Koniks in this reserve, as assessed by the protocols used, was scored high or very high. Although the body condition was lower after the shortage of quality forage in the winter, this is a natural cycle that allows Koniks to survive in the climatic conditions of North East Europe. Our study involved animals that are reproductively active and therefore require more energy. To prevent the metabolic issues typical to native horse breeds, this study should be repeated on more obesity-prone castrated male horses and non-lactating mares. Finally, it should be highlighted that welfare assessment protocols do not replace regular inspections of horses by owners.

## Figures and Tables

**Table 1:** The indicators used in the final assessment of Koniks including the body condition scores proposed by [35] (BCS-H), WAT [11], and IFCE/INRAE [8] protocols.

Welfare Indicator	Description	Scale
BCS-H	From “poor” to “extremely fat”	1–9
**From WAT template**		
BCS-WAT	Poor or very thin and fat or extremely fat/thin or moderately thin/moderate to fleshy	0/1/2
Environment: Water availability	No water detected/pooling water/fresh free-running stream, spring or lake	1/2/3
Environment: Human disturbances/ease of movement	Presence of bikers, dogs, runners, campers/people not inhibiting the movement/no people, dogs, bikes present	1/2/3
Environment: Hazard fencing, man-made hazards	Hazards that inhibit the pony’s ability to move freely/Hazards can be avoid/no hazards present	1/2/3
Environment: Resting comfort	No clean, dry area to rest—muddy and wet/clean, dry area to lay down or rest	1/2/3
Environment: Thermal comfort	No shelter or shade and/or the pony is shivering or sweating/the pony has no shelter or shade but is not shivering or sweating/free access to shelter or shade and/or neither shivering nor sweating	1/2/3
Health: Mobility	Immobile of severely impaired mobility, the pony is unable to stay with the herd/walking with visible signs of difficulty, but the pony can stay with herd/no signs of abnormality	0/1/2
Health: Skin/mane/tail condition	Missing or broken main or tail/normal mane and tail	1/2
Health: Skin/coat condition	Coat patchy or uneven, alopecia, winter coat out of season/no dully or dry coat, no hair loss	1/2
Health: Ocular discharge	Discharge and partial or complete closure of the eye, with or without swelling/discharge with eye open down the cheek/normal eye with no discharge	0/1/2
Health: Nasal discharge	Nasal discharge present/no nasal discharge”	1/2
Health: Coughing	At least one cough/no cough	1/2
Health: Wounds and swellings	Open wound > 7 cm, green or yellow discharge/healing wounds <7 cm/no wounds or swollen areas	0/1/2
Behaviour: Social contact	Solitary, no other ponies visible/other ponies present	1/2
Behaviour: Human avoidance distance	Pony moves away when the assessor is more than 9 m away/pony moves away when the assessor is less than 9 m away/pony does not move away, the assessor must stop a 3 m	1/2/3
**From IFCE/INRAE protocol**	**Description**	**Scale**
Stereotypies	Present at least once per ten 3-s sampling points in 5 min of observation/Absent in all ten 3-s sampling points in 5 min of observation	0/1
“Withdrawn” posture	Recorded in five consecutive sampling points in 5 min of observation/Not recorded in five consecutive sampling points in 5 min of observation	0/1
Aggression toward humans	Present at least once per ten 3-s sampling points in 5 min of observation/Absent in all ten 3-s sampling points in 5 min of observation	0/1
Human avoidance	“Avoidance”/“No avoidance”	0/1
Ears back position	Present in more than two successive 3-s sampling points in 5 min of observation/Present in two or fewer successive 3-s sampling points in 5 min of observation	0/1
Coughing	Present/Absent	0/1
Thermal stress	Present/Absent - In the heat, more than three of the following signs: dilated nostrils, increased respiratory rate (more than 24 respiratory movements/minute), increased range of respiratory movements, head bobbing, apathy, heavy sweating, sunburn (particularly in animals fair-skinned, on the nose or exposed areas of the skin) - In the cold, more than three signs among the following: decrease in frequency respiratory (fewer than six respiratory movements/minute), shallow breathing, shivers, apathy, animals huddled.	0/1
Coat condition	Dry, dull, rough coat/Soft, smooth, homogeneous and shiny coat	0/1
Injuries	Present/Absent Any alteration of the surface integument greater than 2 cm^2^ or more than 4 cm long was used to define the presence of lesions	0/1
Joint swollen	Present/Absent At least one swollen joint	0/1
Hooves condition	Poor/Intermediate/Good	0/0.5/1
Occular discharge	Present/Absent	0/1
Nasal discharge	Present/Absent	0/1
Other discharges (genital, anal)	Present/Absent	0/1
Lameness	The horse is either showing an abnormal gait, can not weight bear on one limb, can not stand or can not move/The horse can weight bear on all four limbs equally and completely both at rest and in walk	0/1
Possiblity for social interactions	No possibility for physical or visual social contact/Possibility for physical or visual social contact, but not engaging in this/Physical or visual social contact with at least one other	0/0.5/1
Resting zone comfort	No clean and dry area for lying/A clear and dry area is not present	0/1
Water—availability	Present/Absent	0/1
Water—cleanliness	Dirty/Partly dirty/Clean	0/0.5/1

0: non-satisfactory, 0.5: intermediary, 1: Satisfactory.

**Table 2:** Descriptive statistics and the effect of season on BCS-H and WAT welfare indicators. Significant effects (*p* < 0.05) were indicated in bold font.

Welfare Indicator	2022	2023		
	Mean ± SD; Minimum–Maximum		S	*p*
BCS-H	5.63 ± 1.01; 2–6	3.84 ± 1.17; 2–7	28	**0.03**
BCS-WAT	1.58 ± 0.61; 0–2	1.11 ± 0.57; 0–2	22.5	**0.01**
Environment: Water availability	2.00 ± 0.00; 2–2	2.00 ± 0.00; 2–2	NV	NV
Environment: Human disturbances/ease of movement	2.79 ± 0.63; 1–3	3.00 ± 0.00; 2–3	1.50	0.50
Environment: Hazard fencing, man-made hazards	2.79 ± 0.63; 1–3	2.68 ± 0.23; 3–3	3.50	0.76
Environment: Resting comfort	3.00 ± 0.00; 3–3	3.00 ± 0.00; 3–3	NV	NV
Environment: Thermal comfort	3.00 ± 0.00; 3–3	3.00 ± 0.00; 2–3	NV	NV
Health: Mobility	2.00 ± 0.00; 2–2	2.00 ± 0.00; 2–2	NV	NV
Health: Skin/mane/tail condition	2.00 ± 0.00; 2–2	1.95 ± 0.23; 1–2	1.50	0.50
Health: Skin/coat condition	0.79 ± 0.42; 1–2	1.89 ± 0.31; 1–2	2.50	0.62
Health: Ocular discharge	1.95 ± 0.23; 1–2	1.95 ± 0.23; 1–2	NV	NV
Health: Nasal discharge	1.89 ± 0.31; 1–2	2.00 ± 0.00; 2–2	1.50	0.50
Health: Coughing	2.00 ± 0.00; 2–2	1.95 ± 0.23; 1–2	0.50	1.00
Health: Wounds and swellings	1.95 ± 0.23; 1–2	1.95 ± 0.23; 1–2	0.50	1.00
Behaviour: Social contact	2.00 ± 0.00; 2–2	1.95 ± 0.23; 2–2	0.50	1.00
Behaviour: Human avoidance	3.00 ± 0.00; 3–3	3.00 ± 0.00; 3–3	NV	NV

NV: no variability; S: signed rank test statistics.

**Table 3:** Descriptive statistics and the effect of season on IFCE/INRAE welfare indicators.

Welfare Indicator	2022	2023		
	Mean ± SD; Minimum—Maximum		S	*p*
Stereotypies	1.00 ± 0.00; 1–1	1.00 ± 0.00; 1–1	NV	NV
Withdrawn posture	1.00 ± 0.00; 1–1	1.00 ± 0.00; 1–1	NV	NV
Aggression toward human	1.00 ± 0.00; 1–1	1.00 ± 0.00; 1–1	NV	NV
Human avoidance	1.00 ± 0.00; 1–1	1.00 ± 0.00; 1–1	NV	NV
Coughing	1.00 ± 0.00; 1–1	0.95 ± 0.23; 0–1	0.50	1.00
Thermal stress	1.00 ± 0.00; 1–1	1.00 ± 0.00; 1–1	NV	NV
Coat condition	0.95 ± 0.16; 0.5–1	0.84 ± 0.37; 0–1	1.00	1.00
Injuries	0.97 ± 0.11; 0.5–1	0.95 ± 0.23; 0–1	0.50	1.00
Joint swollen	1.00 ± 0.00; 1–1	1.00 ± 0.00; 1–1	NV	NV
Hooves condition	0.88 ± 0.28; 0–1	0.91 ± 0.19; 0.5–1	0.00	1.00
Ocular discharge	0.97 ± 0.11; 0.5–1	0.97 ± 0.11; 0.5–1	0.00	1.00
Nasal discharge	0.97 ± 0.11; 0.5–1	1.0 ± 0.0; 1–1	0.50	1.00
Other discharges	1.0 ± 0.0; 1–1	1.0 ± 0.0; 1–1	NV	NV
Lameness	1.0 ± 0.0; 1–1	1.0 ± 0.0; 1–1	NV	NV
Possiblity for social interactions	1.0 ± 0.0; 1–1	1.0 ± 0.0; 1–1	NV	NV
Resting comfort	1.0 ± 0.0; 1–1	1.0 ± 0.0; 1–1	NV	NV
Water—availability	1.0 ± 0.0; 1–1	1.0 ± 0.0; 1–1	NV	NV
Water—cleanliness	0.5 ± 0.0; 0.5 [0.5; 0.5]	0.5 ± 0.0; 0.5 [0.5; 0.5]	NV	NV

1: satisfactory, 0.5: intermediate, 0: unsatisfactory; NV: no variability; S: signed rank test statistics.

## Data Availability

The data presented in this study are available on request from the corresponding author. The data are not publicly available due to their sensitive nature.
